# Receptor-Mediated NETosis on Neutrophils

**DOI:** 10.3389/fimmu.2021.775267

**Published:** 2021-11-04

**Authors:** Tao Chen, Yanhong Li, Rui Sun, Huifang Hu, Yi Liu, Martin Herrmann, Yi Zhao, Luis E. Muñoz

**Affiliations:** ^1^ Department of Rheumatology and Immunology, West China Hospital, Sichuan University, Chengdu, China; ^2^ Rare Diseases Center, West China Hospital, Sichuan University, Chengdu, China; ^3^ Institute of Immunology and Inflammation, Frontiers Science Center for Disease-Related Molecular Network, West China Hospital, Sichuan University, Chengdu, China; ^4^ Department of Internal Medicine 3 - Rheumatology and Immunology, Friedrich-Alexander-University Erlangen-Nürnberg (FAU) and Universitätsklinikum Erlangen, Erlangen, Germany

**Keywords:** neutrophil extracellular traps, chemokine receptor, Fc receptors, complement receptors, pattern recognition receptors

## Abstract

Neutrophil extracellular traps (NETs), a web-like structures containing chromatin, have a significant role in assisting the capture and killing of microorganisms by neutrophils during infection. The specific engagement of cell-surface receptors by extracellular signaling molecules activates diverse intracellular signaling cascades and regulates neutrophil effector functions, including phagocytosis, reactive oxygen species release, degranulation, and NET formation. However, overproduction of NETs is closely related to the occurrence of inflammation, autoimmune disorders, non-canonical thrombosis and tumor metastasis. Therefore, it is necessary to understand neutrophil activation signals and the subsequent formation of NETs, as well as the related immune regulation. In this review, we provide an overview of the immunoreceptor-mediated regulation of NETosis. The pathways involved in the release of NETs during infection or stimulation by noninfectious substances are discussed in detail. The mechanisms by which neutrophils undergo NETosis help to refine our views on the roles of NETs in immune protection and autoimmune diseases, providing a theoretical basis for research on the immune regulation of NETs.

## 1 Introduction

Faced with daily exposure to various pathogens, humans rely heavily on the innate immune system as a first responder to intruders. Neutrophils are the most abundant and fastest responding innate immune effector cells. These cells play a central role in innate immunity by acting when inflammation occurs and then subside (1). When pathogens invade the body, neutrophils kill them through three major strategies: phagocytosis, degranulation and the release of neutrophil extracellular traps (NETs) (2). NETs are extracellular, web-like structures consisting of DNA-histone complexes and decorated by a set of neutrophil granule proteins, such as cathelicidin, cathepsin G, myeloperoxidase (3) and neutrophil elastase (NE) (4–6). These extracellular structures trap pathogenic microbes, preventing them from spreading, and ensure a high local concentration of antimicrobial agents to degrade virulence factors and kill microorganisms (7). However, molecules released during NET formation can often become autoantigens involved in the pathogenesis of autoimmune diseases. For example, citrullinated proteins (such as histone H3, histone H4 and vimentin) are new neoepitopes for anti-citrullinated protein antibodies (ACPA) in rheumatoid arthritis (RA); NET-derived extracellular nucleic acids and dsDNA are the targets of systemic lupus erythematosus (SLE) autoantibodies; NET-associated MPO and proteinase 3 (PR3) enzymes are major autoantigenic targets of anti-neutrophil cytoplasmic antibody (ANCA)-associated vasculitis (AAV) (8–10). However, although NETs play a very important role in the occurrence, development and resolution of gout induced by monosodium urate (MSU) crystals, there are currently no reports of related autoantibody abnormalities, and the specific mechanism remains to be further explored (11).

Receptors in the innate immune system are specialized surface proteins that recognize foreign particles and pathogenic bacteria. Receptors can detect or perceive the type of foreign pattern during infection, so these are also called pattern recognition receptors (PRRs) (12). Following ligand recognition, the triggered intracellular signaling cascade leads to the transcription and/or secretion of inflammatory mediators, coordinating the elimination of pathogens and infected cells. Neutrophils are at the forefront of identifying and subsequently killing invading pathogens. These immune cells express a vast repertoire of PRRs, from members of the Toll-like receptor (TLR) family to dendritic cell-associated C-type lectin 1 (Dectin-1) molecules that specifically recognize a large number of glycoproteins (13). Neutrophils are also stimulated by a variety of substances, including opsonized particles, immune complexes and chemokines. Binding to PRRs leads to degranulation, production of reactive oxygen species (ROS), and NETs and ultimately to the elimination of invasive pathogens (14). Therefore, it is of major importance to understand the specific mechanisms of neutrophil activation that facilitate the identification of therapeutic targets for abnormal neutrophil activation in disease.

In this review, we summarize the different receptors expressed on neutrophils and the formation of NETs, which first requires the activation of neutrophil receptors. We also describe in detail the receptors known to be expressed on human neutrophils involved in NET formation and discuss their specific mechanisms for participation in the release of NETs.

## 2 NETosis

Neutrophils release NETs *via* a multistep process called NETosis. To date, two key mechanisms by which neutrophils release NETs in host defense are known as suicidal NETosis and vital NETosis (15). The specific mechanisms that regulate these forms of cell death have not yet been completely clarified.

Suicidal NETosis was first observed in response to phorbol-12-myristate-13-acetate (16), an effective activator of multiple signaling pathways in neutrophils (7). Fuchs et al. used detailed *in vitro* cell imaging technology to define NET release as the NOX-dependent cell death process. This form of suicide is a step-by-step progression after chromatin decondensation, nuclear swelling, overflow of nucleus to cytoplasm, and finally membrane perforation mixing of nucleic acids and granular proteins (17). Suicidal NETosis involves the triggering of nicotinamide adenine dinucleotide phosphate (NADPH) oxidase (NOX)-dependent pathways, the generation of ROS, and receptor-interacting protein kinase/mixed lineage kinase domain-like-mediated signals (18). Briefly, the activation of receptors increases calcium ions and stimulates the activity of protein kinase C (PKC) and NOX, leading to the formation of ROS. Under ROS, activation of MPO not only participates in the depolymerization of chromatin and the rupture of the nuclear membrane, forming the main components of NETs with other proteins and DNA, but also further activates NE, which promotes the transfer of NE from the cytoplasm to the nucleus, cleaving chromatin and releasing it into the cytoplasm. In addition, MPO also contributes to the decondensation of chromatin, but the enzymatic activity of MPO is not required at this stage. Interestingly, in this process, the peptidyl arginine deiminase 4 (PAD4) enzyme is also activated (19). Subsequently, the combined action of PAD4, NE, and MPO results in citrullination of histone H3 and subsequent chromatin decondensation, discharging into the extracellular space and resulting in neutrophil death. This process is also called NOX-dependent NETosis.

In contrast to the relatively slow suicidal NETosis pathway, vital NETosis involves the rapid release of NETs stimulated by microorganisms, activated platelets (PLTs), or complement proteins through neutrophil surface receptors, which appears to be independent of NOX activity (20, 21). In this pathway, the main feature of vital NETosis is activation by Ca^2+^-activated PAD4, citrullinating H3 and participating DNA decondensation with the cooperation of MPO and NE (22). Next, the nucleus loses its classical lobular shape, and decondensed chromatin is expelled to form NETs. Under this condition, the release of DNA, granular proteins and histones occurs with the formation and extrinsic distribution of vesicles, so the cell is still alive and has the capacity to perform cellular functions, such as cell migration (5). The influx of Ca^2+^ is considered to require the participation of mitochondrial ROS (mitoROS). However, it is still controversial whether mitoROS are involved in NOX-independent NETosis.

## 3 Receptors That Trigger NETosis

### 3.1 Pattern Recognition Receptors

PRRs are essential for detecting invading pathogens and initiating innate and adaptive immune responses. The ligands of these receptors consist of specific pathogen-related molecular patterns (PAMPs) of microbial molecules and damage-related molecular patterns (DAMPs) exposed on the surface of damaged cells (12). There are multiple families of PRRs, including membrane-associated TLRs, C-type lectin receptors (CLRs), nucleotide-binding oligomerization domain-like (23) receptors (NLRs) and RIG-I-like receptors (RLRs) (12). Among these, TLRs, CLRs and NLRs have been reported to be involved in NETosis.

#### 3.1.1 Toll-Like Receptors

TLRs play a vital role in host cell recognition and responses to microorganisms. To date, 11 TLR family members have been identified, of which TLR1, TLR2, TLR4, TLR5, TLR6 and TLR11 are located on the cell surface and primarily recognize the microbial membrane, while the other members are located in endosomes/lysosomes and recognize foreign nucleic acids (24). As the first line of defense, these receptors play key roles in pathogen invasion, innate immune responses and antigen-specific adaptive immunity (25). Human neutrophils express all TLRs except TLR3 (26). Neutrophils mainly recognize PAMPs through their TLRs, thereby triggering responses to invading pathogens. TLR activation leads to important cellular processes, including ROS generation, degranulation, NET formation, and cytokine production (27). When signal transduction is dysregulated, chronic inflammation might result. In chronic inflammation, NET imbalance maybe can be considered to one of the main factors.

In recent years, a majority of TLRs have been identified to participate in NETosis, as these receptors can specifically recognize a variety of pathogenic microorganisms, such as viruses, bacteria, parasites and fungi. Giving examples, the smallest prokaryotic microorganism in the biological world, *Mycoplasma agalactiae (M. agalactiae)*, was the first reported to induce NET release *via* the TLR2 signaling pathway, but its mechanism is not yet clear (28). Additionally, in the natural immune confrontation between fungus and host, the receptors TLR2 and TLR4 are essential for ROS-dependent NETosis induction by *Fonsecaea pedrosoi* (*F. pedrosoi*) (29). Regarding parasites, *Eimeria bovis* (*E. bovis*) can increase TLR2 and TLR4 expression on the PMN and induce TLR2/4-dependent NF-κB activation, resulting in NETosis (30). Regarding bacteria, *Streptococcus suis serotype 2 (SS2)* can be recognized by TLR2 and/or TLR4, initiating NETosis in a NOX - dependent manner. And, blocking TLR4 signaling could further inhibit the activation of ERK1/2 without p38 MAPK (31). Another kind of bacteria, *Wolbachia endobacteria* (*W. endobacteria*), interacts with TLR2/6 to trigger NETosis through direct ligation of *Wolbachia* lipoprotein (32). Moreover, various types of viruses reported to be involved in NETosis are common. For instance, activation of PLT receptor C-type lectin member 2 (CLEC2) by dengue virus (DV) (33) or H5N1 influenza virus (IAV) enhances NETosis and proinflammatory cytokine production *via* TLR2 (34), while respiratory syncytial virus (RSV) F protein leads to NET production dependent on TLR4 activation, NOX-derived ROS production and ERK and p38 MAPK phosphorylation (35). Additionally, human immunodeficiency virus 1 (HIV-1) is captured and killed in NETs formed by neutrophils using TLR7 and TLR8 to recognize viral nucleic acids (36), and chikungunya virus (CHIKV) induces NETosis through a mechanism dependent on TLR7 activation and ROS generation (37). These pathogenic microorganisms are captured and killed by activating TLRs to participate in neutrophil-mediated NETosis. Although the specific mechanism needs to be further explored, the generation of ROS and the activation of the ROS-dependent NETosis pathway play irreplaceable roles in this process.

In addition to pathogens, DAMPs are also involved in NET release by triggering TLRs. During liver ischemia/reperfusion (I/R) injury, histones and high-mobility group box 1 (HMGB1) released from damaged hepatocytes function as DAMPs to promote PAD4 activation *via* TLR4 and TLR9 signaling pathways, which subsequently activate NETosis (38). Oxidized low-density lipoprotein (oxLDL) can induce NETs by TLR4 and TLR6 *via* the ROS-dependent pathway (39). In patients with antiphospholipid syndrome (APS), anti-β2 GPI/β2 GPI antibodies induce NETosis to promote thrombogenesis *via* the TLR4/MyD88/MAPK signaling pathway (40). In addition, activated PLTs can induce NETs in a TLR4-dependent manner, promoting the trapping of bacteria within blood vessels in septic patients (41). Moreover, mitochondrial DNA (mtDNA) activates neutrophils *via* the cyclic GMP-AMP synthase (cGAS) and TLR9 pathways to induce NETosis (42).

#### 3.1.2 NOD-Like Receptor

NLRs are cytosolic receptors that provide a second line of defense against pathogen invasion. NOD1 and NOD2 are two well-characterized NLRs belonging to the NLRC subfamily that recognize components of bacterial peptidoglycan. Other NLRs, such as NOD-like receptor family pyrin domain containing 1 (NLRP1), NLRP3 and NLRC4, are activated by a number of different pathogens and damage signals and oligomerize to form multiprotein inflammasome complexes.

There are very few reports on NLR family involvement in NETs, and the first report was in 2019 by Alyami and his team. They found that Fusobacterium nucleatum upregulated NOD1 and NOD2 to activate neutrophils in a time-dependent manner and induce strong NETosis. Furthermore, employing CRISPR/Cas9 knockout of NOD1/NOD2 in HL-60 cells and inhibitors of NOD signaling, *Fusobacterium nucleatum (F. nucleatum)* has been confirmed to mediate NETosis through the activation of the PAD4 enzyme and the release of MPO and NE (43). In addition to NOD1 and NOD2, NLRP3 has recently been reported to be involved in NETosis, but its role in NET formation is rather complicated. While there is no specific mechanism to show the connection between NLRP3 and NETosis, the assembly of NLRP3 inflammasomes requires PAD4 to participate in the rupture of nuclear and plasma membranes. PAD4 activity and rupture of nuclear and plasma membranes are the key steps in the formation of NETs. This phenomenon was verified in mouse and human neutrophils, and it was found that pharmacological inhibition of NLRP3 also reduced NETosis and that NLRP3 deficiency resulted in a lower density of NETs in thrombi produced in a stenosis-induced mouse model of deep vein thrombosis (44). Considering the clinical importance of excessive IL-1β and NET generation, PAD-dependent regulation of NLRP3 protein levels could be an important mechanism in inflammasome-driven diseases. Targeted blockade of NLRP3 may reduce the nocive effects of NETs. NLRP3 is expected to become a new target for the treatment of several diseases, including acute gouty arthritis, thrombosis and type II diabetes.

#### 3.1.3 C-Type Lectin Receptors

CLRs compose a transmembrane protein family, with members having at least one C-type lectin-like domain (CTLD) at the cell surface. Classical CLRs are proteins that bind various carbohydrate moieties in a calcium-dependent manner through conserved residues within the CTLD. Immune cells, including all myeloid cells and lymphocytes, express various CLRs. Some CLRs, such as L-selectin, Galectin-1 (Gal-1), macrophage inducible C-type lectin (Mincle), myeloid inhibitory C-type lectin (MICL), Dectin 1 and C-type lectin-2 (Dectin 2), are commonly expressed on neutrophils (45). These CLRs can directly recognize microbial membrane glycans and activate innate immunity by triggering inflammatory cytokine secretion, NET formation and the antibacterial response in neutrophils (46).

Interestingly, unopsonized *Candida albicans (C. albicans)* yeast-induced NET formation requires the Dectin 2-mediated Syk-Ca^2+^- PAD4 signaling pathway through a NOX-independent pathway to restrain the spread of C. albicans from the peritoneal cavity to the kidney (47). However, opsonized *C*. *albicans* induced NETosis *via* NOX to capture and kill the pathogen (48). Protectively, the host regulates NETosis through dual mechanism-dependent and independent NOX pathways, which quickly fight pathogenic microorganisms and exert antibacterial effects. In addition, CLEC2 and C-type lectin member 5A (CLEC5A) are critical in microbe-induced NET formation, such as that caused by dengue virus or H5N1 influenza virus (34, 49). Moreover, CLEC5A is needed for optimal ROS production, NET formation and other immune responses to Listeria monocytogenes in mice (50). Thus, CLR-mediated NETosis pathways are potent endogenous danger signals, and blocking C-type lectins may be a promising strategy to inhibit virus-induced NETosis and cytokine storm. Another CLR, Mincle, can mediate NET formation *via* modulation of autophagy without being affected by ROS, which is a major discovery in this field (51). Mincle is considered a therapeutic target that selectively inhibits NETs without affecting ROS generation.

Interestingly, in addition to participating in the formation of NETs, CLRs can also prevent NET release. NET release is dependent on neutrophils sensing the size of microorganisms and selectively releasing NETs in response to large pathogens, such as *C. albicans* hyphae and *Mycobacterium bovis (M. bovis)*, but not small yeasts or single bacteria. As a size sensor for neutrophil phagocytosis of microorganisms, Dectin-1 can prevent NETosis by inhibiting the transport of NE to the nucleus to cleave chromatin (52). Therefore, this regulatory mechanism underlying the size-dependent release of NETs *via* TLR signaling allows selective implementation of neutrophil antibacterial strategies to eliminate fungal infections while minimizing tissue damage.

### 3.2 Complement Receptors

Complement receptors (CRs), expressed notably on myeloid and lymphoid cells, exert critical functions in the modulation of innate and adaptive immune responses, which interact specifically with complement factors to eliminate antigens from the circulation, clear apoptotic cells and control certain bacterial infections (53). However, recent studies have clearly demonstrated the pathophysiological importance of the complement system in NET-mediated autoimmune diseases. To date, the most commonly identified CRs that contribute to neutrophil NET release are CR1 (CD35), CR3 (Mac-1 or CD11b/CD18), CR4 and CR5.

Aggregatibacter actinomycetemcomitans (A. actinomycetemcomitans) and Actinomyces viscosus (A. viscosus) can induce NETosis in neutrophils through CR1 (54). By interacting with CR3, Aspergillus fumigatus (A. fumigatus) and Staphylococcus aureus (S. aureus) activate downstream NOX to induce NET formation (54, 55). In addition to bacteria, some viruses seem to be recognized by neutrophils via CRs. For instance, work on Hantaan virus (HTNV) has indicated that CR3 and CR4 are critical for NET formation, relying on a ROS-dependent pathway (56). Emerging data indicate that during coronavirus disease 2019 (COVID-19), severe acute respiratory syndrome coronavirus 2 (SARS-CoV-2) triggers complement activation by interacting with C3, which leads to C3a, C5a, and sC5b-9 (TCC) generation. Subsequently, C3a might activate PLTs, while C5a and PLT-derived thrombin induce both neutrophil tissue factor (TF) expression and NETs carrying active TF (57). This important discovery provides the basis for the key roles of complement and NETs in COVID-19 immunothrombosis. Therefore, these studies support the use of complement or NETosis inhibition to fight viral infections and help eliminate the corresponding complications. Yeasts are eukaryotic, single-celled microorganisms classified as members of the fungal kingdom, which are also reported to fight against host neutrophils to form NETs. Histoplasma capsulatum var. capsulatum (H. capsulatum) yeast is a dimorphic fungus with a global distribution that causes histoplasmosis. H. capsulatum yeast induces NETosis through an oxidation mechanism that is dependent on ROS and the Src and Syk kinase pathways by targeting CR3 (58). Another pathogenic yeast opsonized C. albicans, one of the top leading causes of healthcare-associated bloodstream infection, can interact with CR3 to trigger NETs by activating downstream Syk-dependent NADPH oxidase (59). Based on these observations, by releasing NETs, neutrophils create a favorable extracellular microenvironment for yeast trapping and killing, which may explain why people with strong immune capabilities can resolve infections or develop subclinical symptoms, while immunosuppression may cause a disseminated disease.

In addition, high expression of C5aR1 was confirmed to be associated with the NET marker MPO-DNA in a cohort of patients with stable coronary artery disease (60), indicating that there is a clinically relevant interaction between complement activation and NETosis (61). Moreover, ICs have also been confirmed to participate in activating neutrophils by binding to surface CR3 on neutrophils. The specific mechanism is still unclear, but it has been confirmed that the activation of the receptor CR3 by ICs relies on the ROS-dependent NETosis pathway (62).

### 3.3 Fc Receptors

Fc receptors (FcRs) are expressed on various immune cells and can initiate an immune response at the initial antigen presentation step by facilitating IC uptake to drive cellular and humoral immune responses. Human neutrophils constitutively express two antibody receptors that are members of the FcR family recognizing IgG molecules, namely, FcγRIIa (CD32a) and FcγRIIIb (CD16b) (63).

Recent research has indicated that ICs are capable of inducing NETosis. In one report, FcγRIIIb promoted endocytosis of ICs, and FcγRIIa mediated the activation of NETosis (23). However, another report suggested that FcγRIIa promoted phagocytosis and that only FcγRIIIb participated in the induction of NET formation, triggering NETosis *via* the FcγRIIIb cross-linking TAK1-dependent MEK/ERK signaling pathway (64, 65). Therefore, it is not possible to determine which receptor plays the primary role; furthermore, their interaction may be essential in NET formation. In addition, FcRs also seem to take part in NETosis during bacterial infections. The results presented for neutrophils in contact with opsonized S. aureus or hypervirulent Klebsiella pneumoniae (hvKp) suggested that activation of FcRs could enhance the release of NETs (54, 66). Moreover, coating bacteria with IgA could also enhance NETosis against viral pathogens *via* FcαIR on neutrophil signaling through a TLR-independent NOX-dependent pathway (67). Neutrophils expressing FcRs are the first to respond to sites of injury or infection, and IgA virus ICs potentiate NETosis to trap and inactivate viruses, consistent with an antiviral function. NETosis plays a role in protecting the body but can also have pathogenic consequences when poorly regulated.

### 3.4 Chemokine Receptors

Chemokine receptors, which are seven-transmembrane G protein-coupled receptors, are recognized as the most critical mediators for recruiting neutrophils during inflammation. Furthermore, they are also considered to regulate the short lifetime of neutrophils, mobilizing these cells from the bone marrow to the blood to execute immune effects and driving homing to the bone marrow for apoptosis and clearance (68). Certainly, chemokine receptors are also important mediators of neutrophil effector functions, such as oxidative burst and inflammatory cytokine production, degranulation, and NETosis. Among several chemokine receptors, only CXCR1, CXCR2 and CXCR4 have been identified to participate in NET formation.

It’s well known that cholesterol crystals act as danger signals in atherosclerosis (AS), which triggered neutrophils to release NETs. Then, NETs activate macrophages and Th-17 cells, amplifying the recruitment of immune cells in atherosclerotic plaques (69). The role of CXCR2 in the activation of NETosis was confirmed in atherosclerosis (AS), as well as diffuse large B-cell lymphoma (DLBCL) (70, 71). This receptor plays notable roles in aggravating AS and DLBCL progression *in vivo*, cooperating with its ligand IL-8 to release NETs *via* Src kinase, extracellular signal-regulated kinase, and p38 mitogen-activated protein kinase (MAPK) signaling. Moreover, Ca(2+) signaling contributes to p38 MAPK activation (72). The effect of Ca 2+ signaling inhibitors has also been reported to reduce the production of IL-8 (73). So far, although there is no evidence that Ca2+ is directly related to IL8-induced NETosis, previous research supports our conclusion that Ca2+ also plays an important role in it. Moreover, CXCR2 can also send out a signal to recruit neutrophils by cooperating with P-selectin glycoprotein ligand-1 (PSGL-1) to induce NET formation, which further enhances deep vein thrombosis (74). Interestingly, in a recent study on circadian regulation of neutrophil NETosis, it was found that CXCR2 not only recruits neutrophils to local sites of inflammation but also participates in regulating the circadian rhythm to change the NET-forming capacity by disarming the process involving the neutrophil proteome (75). In addition, CXCR4 was identified as an important surface recognition molecule in patients suffering from severe malaria, in whom it functions by releasing macrophage migration inhibitory factor (MIF), which in turn causes NET formation (3).

NETosis has also been reported to be involved in cancer progression, metastatic dissemination, and tumor-associated complications, such as thrombosis and kidney-associated damage (76). CXCR1 and CXCR2 have been proven to be the major mediators of tumors exhibiting ELR+ CXCL chemokine-promoted NETosis (77). Significantly, the study also clarified the protective effect of NETs, which can coat and shield tumor cells against cytotoxic effects mediated by CD8+ T cells and NK cells (77). This protective mechanism is mainly mediated by NETs, degrading extracellular DNA and reducing toxicity, which reduced the interaction between effector cells and target cells. In this sense, NETs are a double-edged sword in tumors that can be responsible not only for capturing metastatic emboli in the bloodstream but also for protecting immune cells from NK cell-mediated cytotoxic attacks. However, the DNA component of NETs (NET-DNA) can promote cancer metastasis through coiled-coil domain containing protein 25 (CCDC25), a specific DNA sensor. After extracellular amino acids ^21–25^ are induced by NET-DNA, CCDC25 interacts with integrin-linked kinase through its intracellular C-terminus and triggers the β-parvin-RAC1-CDC42 cascade reaction, which induces bone rearrangement and directional migration of tumor cells. In an *in vivo* mouse model, targeting CCDC25 reduced NET-mediated distal metastasis (78). However, the functional role and clinical importance of NET-DNA in metastasis in cancer patients are still unclear.

### 3.5 Other Neutrophil Receptors

In addition to the classic neutrophil receptors, other receptors have been reported to mediate NETosis. For instance, NETs have been confirmed to be involved in COVID-19 pathophysiology. angiotensin converting enzyme 2 (ACE2), with the simultaneous involvement of human transmembrane protease, serine 2 (TMPRSS2), interacts with the S protein of SARS-CoV-2, which helps SARS-CoV-2 enter the host cell and induce PAD4-dependent NETosis, leading to fatal respiratory failure associated with an excessive inflammatory response (79). The increases in MPO-DNA and histone-DNA complexes observed in the blood indicate that the release of NETs is involved in the early host response to SARS-CoV-2 infection. Therefore, the blood level dynamics of NETs can predict the severity of COVID-19 in a larger population (80–82). Moreover, NETs have potentially harmful effects on lung epithelial cells and endothelial cells, so synthesis inhibitors or fragmentation promoters will become prognostic targets for COVID-19 in the future and lead to improvements in multiple-organ damage prevention in the clinic.

Ca^2+^ signaling, a universal intracellular messenger, is the key process associated with neutrophil functions, such as the regulation of proinflammatory functions, the formation of NETs and the secretion of cytokines (83). An intracellular Ca^2+^ concentration ([Ca^2+^]i) capable of inducing PAD activation is crucial in NETosis formation. Ca^2+^ receptors/channels are divided into six classic pathways: transient receptor potential (TRP), TRP channels, voltage-gated calcium channels, ryanodine receptors, inositol-1,4,5-triphosphate receptors, store-operated Ca^2+^ entry, and mitochondrial calcium uniporters. However, only the calcium-permeable channel transient receptor potential melastatin 2 (TRPM2), a cation channel that senses ROS, has been reported to trigger NETosis by activating the AMPK/p38 MAPK pathway and autophagy machinery in a highly oxidative environment (84). Now that the importance of Ca^2+^ in neutrophil NET formation and cytokine secretion has been emphasized, it is necessary to further explore the mechanism of Ca^2+^-dependent NETosis to provide a theoretical basis for therapies that regulate neutrophil function, such as Ca^2+^ influx inhibitors, to block the formation of NETs and prevent autoimmune and chronic inflammatory diseases.

Recently, Caspase-11, a cytosolic endotoxin receptor, was reported to be involved in the morphological features of NETosis induced by caspase-11/gasdermin D (GSDMD) signaling (85, 86). In the final stage of NET release, neutrophil plasma membrane rupture requires caspase-11 and GSDMD, but it is independent of MPO, NE, and PAD4 (86). During caspase-11-driven NETosis, PAD4-dependent NETs may flow through the GSDMD pore through calcium influx and histone H3 citrullination, but this accompanying process may not be necessary for NETosis. The specific mechanism of NETosis induced by these two processes may occur through different signaling mechanisms converging on the common executioner protein GSDMD in response to different challenges. Therefore, characterizing the functions of Caspase-11 and GSDMD in inducing NETosis could reveal these molecules as previously neglected therapeutic targets in autoimmune inflammatory diseases.

Importantly, NETs play a role in the pathogenesis of periodontitis and RA (87). However, the mechanism of NET information in periodontitis is unclear. Recently, a new signaling pathway was discovered. *Porphyromonas gingivalis* (*P*. *gingivalis*) hijacks a host strategy for disarming pathogens by triggering NETosis through gingipain-dependent cleavage of protease activated receptor-2 (PAR-2), which is the most abundant PAR on human and murine neutrophils (88). Mechanistically triggering NETosis, PAR-2 is activated by cleavage of the extracellular N-terminus at a canonical site (Arg36^#Ser37^), exposing a tethered ligand at the new N-terminal receptor sequence by proteolytically active gingipain depending on NOX activation and ERK-dependent signaling (88). Therefore, under the pathological conditions of excessive NETosis, inhibition of PAR signaling should be regarded as a new treatment in patients with RA and periodontitis.

## 4 Conclusion

NETs are key in coordinating the innate immune response and participating in the elimination of pathogenic microorganisms. The formation of NETs is triggered by receptor-ligand binding events and is regulated by a series of intracellular signaling pathways. However, dysregulated NETs may cause excessive collateral damage to the host, such as inflammation and autoimmune diseases. Therefore, the production of NETs must be strictly regulated to protect the host from pathogens while not causing harmful inflammation and tissue damage. Importantly, the receptors on neutrophils are the key for mediating NETosis.

**Figure 1:**
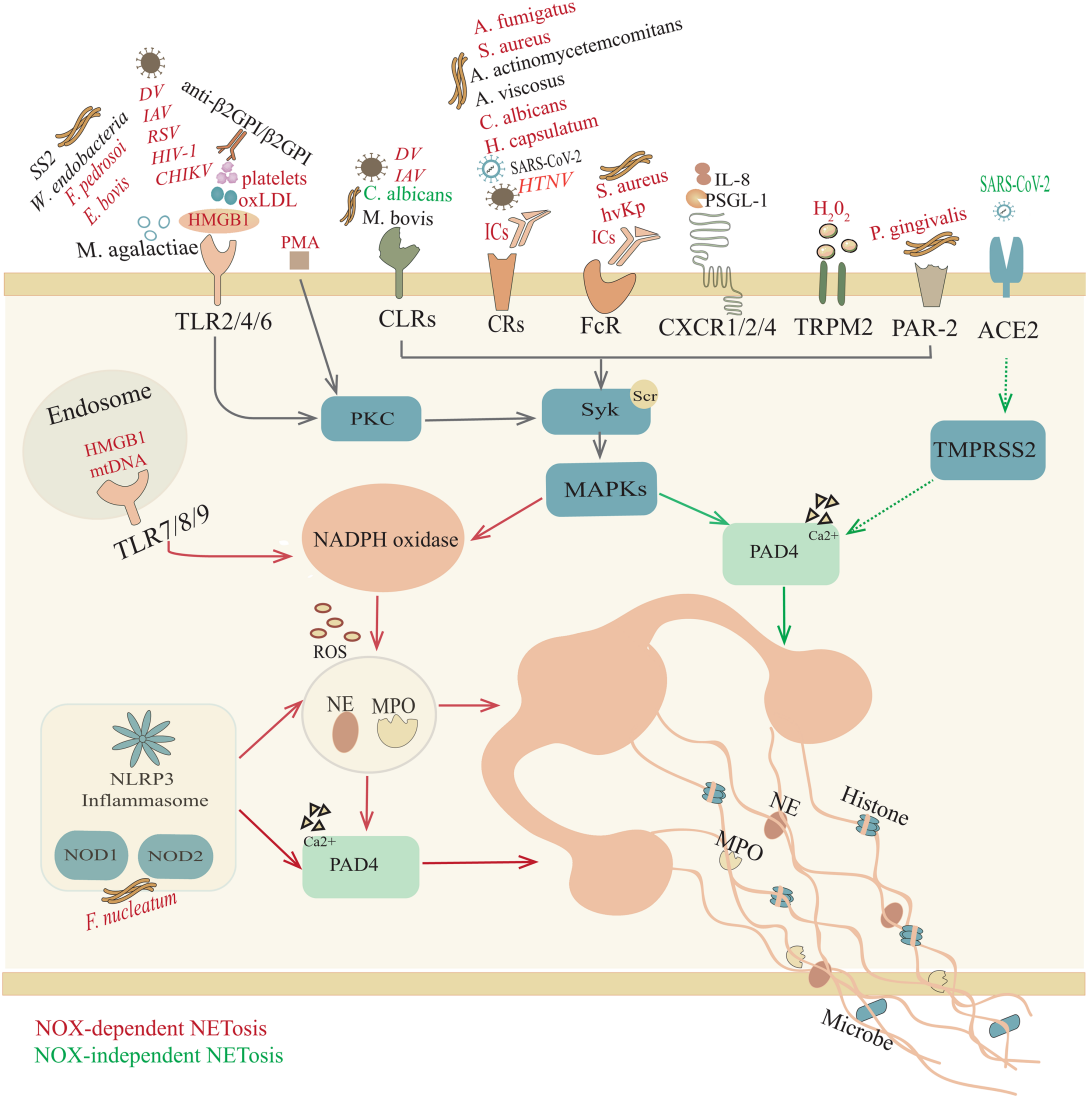
Overview of NETosis. Trigger factors, such as pathogenic microorganisms, ICs, HMGB1, oxLDL, H_2_O_2_, and mtDNA, induce NOX-NETosis. In response to different stimuli, neutrophils participate in a response through their surface receptors, including Toll-like receptors (TLRs); C-type lectin receptors (CLRs); complement receptors (CRs); Fc receptors (FcRs); chemokine receptors (CXCRs); NOD-like receptors (NOD1/2); transient receptor potential melastatin 2 (TRPM2); and protease activated receptor-2 (PAR-2), initiating p38 mitogen-activated protein kinase (MAPK) signaling and producing ROS, which activate MPO, NE and PAD4. Afterward, activated NE and MPO are transferred to the nucleus to promote further unfolding of chromatin, thereby destroying the nuclear membrane. However, in the NOX-independent NETosis pathway, unopsonized *C. albicans* yeast interacts with CLRs (Dectin 2) and SARS-CoV-2 bonds with angiotensin converting enzyme 2 (ACE2) and transmembrane protease serine 2 (TMPRSS2) to activate PAD4, which can lead to histone citrullination and participate in chromatin decondensation. Subsequently, chromatin is released into the cytoplasm, where it is decorated with granules and cytoplasmic proteins. However, for some other microorganisms (*M. agalatiae, SS2, M. bovis, A. actinomycetemcomitans, A. viscosus, SARS-CoV-2)*, IL-8, PSGL-1 and anti-β2GPI/β2GPI, it is still unclear whether the specific mechanism of NET formation requires ROS.

In summary, although we have a certain understanding of the common receptors and signal transduction pathways involved in NET release from neutrophils (**Figure 1**), it is still unclear whether other receptors on neutrophils are involved in NETosis. The work that needs to be continued is better defining how various fields of biology relate to NETosis, including the molecular mechanisms that control NET production and the downstream pathways that lead to NETosis. Weighting the advantages and disadvantages of NETs will ultimately maximize benefits to humans. This work is expected to open a new avenue for current and future research to develop tools to regulate inflammation.

## Author Contributions

TC and YHL wrote the manuscript. TC, RS, HH, and YL generated the figure and assisted with editing. MH and LM edited the manuscript. YZ conceived the idea and supervised the writing process. All authors contributed to the article and approved the submitted version.

## Funding

This work was supported by the National Key Research and Development Program of China (2019YFE0108200), the National Key Research and Development Program of China (2016YFC0906201), the National Natural Science Foundation of China (81771742, 81770101, 81403041, 82001728), Sichuan University postdoctoral interdisciplinary Innovation Fund, 1·3·5 project for disciplines of excellence, West China Hospital, Sichuan University (ZYGD18015, ZYJC18003, ZYJC18024), and China Postdoctoral Science Foundation (2019M663522).

## Conflict of Interest

The authors declare that the research was conducted in the absence of any commercial or financial relationships that could be construed as a potential conflict of interest.

## Publisher’s Note

All claims expressed in this article are solely those of the authors and do not necessarily represent those of their affiliated organizations, or those of the publisher, the editors and the reviewers. Any product that may be evaluated in this article, or claim that may be made by its manufacturer, is not guaranteed or endorsed by the publisher.
